# Supplementary material for: “They organized women who advised me”: Adolescents’ perceptions of Social and cultural influences on mental health during the postnatal period in Malawi: Qualitative study
Source: PLOS Ment Health. 2025 Feb 6;2(2):e0000044. doi: 10.1371/journal.pmen.0000044 (PMC12798599; doi:10.1371/journal.pmen.0000044)
Supplement: Attachment — Submitted filename: Responses to the reviewrs 5.12.24.docx [file pmen.0000044.s001.docx]

Dear Editor,

Thank you for reviewing this manuscript. We have addressed the comments and presented the responses in the table below

| . Reviewers/ editors comments’ | Responses |
| --- | --- |
| If there are ethical or legal restrictions on sharing a de-identified data set, please explain them in detail | There are legal restrictions on data sharing because of the confidentiality agreement we made with participants. Therefore, our data availability is restricted, and we have stated the following  *Curtin University survey office, which manages the Human Research Ethics Committee for researchers, allows limited access to data generated for this study to protect participants' privacy and related confidentiality agreements. However, interested researchers may reach out to the Human Research Ethics Committee (hre@curtin.edu.au), or the chairperson of the Human Research Ethics Committee and the corresponding author, Mrs. Chimwemwe Tembo (chimweptembo@yahoo.co.uk), to request access to data if needed to reproduce the article or review results* |
| Please update your Data Availability statement in the submission form accordingly. | This availability statement is updated accordingly |
| 3. Please provide separate figure files in .tif or .eps format | All figures have been attached separately and in TIF format |
| **Reviewer 1** |  |
| A well-written manuscript. I was eager to know the outcome of the study. The description of each method was precise. It is an adaptable study approach that I would also consider applying in Zambia to assess if the outcomes would be similar or differ. | Thank you for the comments. |
| **Reviewer 2** |  |
| **Abstract** |  |
| The title of the article is not reflective of the study objective and/or the core research question that the authors aim to address in this qualitative study. | Thank you for your comment, we have rephrased it to  “*They Organized Women Who Advised Me”: Adolescents’ perceptions of Social and cultural influences of mental health during the postnatal period in Malawi: Qualitative study* |
| The result section of the abstract needs more clarity on how the findings reflect the research objective, i.e. to explore perceived cultural and social factors influencing mental health among adolescent women.  It would be better if the author omits the Discussion section on the abstract and merged the findings portions into the result section and the conclusion to the conclusion section of the abstract Introduction | *Thank you for your comment. We have deleted the discussion section and merged the findings within the results section****.***  **Lines 27-39**  ***Results***  *Adolescent mothers’ mental health was found to be influenced by interactions between social and cultural factors within all levels of the Social Ecological Model of Health (SEM). Four main themes, health and mental health awareness, relationships, family support, and rite of passage, explain social and cultural influences for adolescent mothers in Malawi.*  ***Conclusions and Recommendations***  *Social and cultural factors influence mental health positively and negatively. Therefore, there is a need for collaborative, coordinated and co-designed efforts to implement integrated, comprehensive, and culturally appropriate interventions at each level of SEM influence to promote adolescents’ mothers’ mental health. Specifically, policies should focus on prevention of early marriages, improved access to reproductive health services, and long-term interventions to promote family involvement in the care of mothers. Health care systems should offer accessible mental health support for adolescent mothers.* |
| In the introduction section author has tried to illustrate the current status of adolescent pregnancy in the region followed by the legal provision and strategies adopted by the government of Malawi to control the issue of child marriage. Then the author moves to the potential consequences of adolescent motherhood and surgically connects it with the postnatal period. The author talks about the importance of seeking mental health support and early identification problems followed by setting the connection between cultural and social factors impacting mental health disorders. o But nowhere in the introduction, the author has specified: what is the nature of the mental health problems that adolescent mothers are experiencing and what are the potential social and cultural factors that have been observed to affect maternal mental health in countries similar to Malawi. Though this is a qualitative study the author could look into some quantitative studies so they could reflect more on the current magnitude of maternal mental health conditions such as antenatal depression, postpartum depression, maternal blues, anxiety, stress, and others in the context of Malawi, Africa, and Asian context which share similar social and cultural attributes.  There is a need to spell out the cultural and social factors that might affect maternal mental health so that international readers can better understand the context in which this study is executed. | Thank you for your comment. we have added more information lines 71 - 89  *In low- and middle-income countries (LMICs) approximately 16 % to 40% of perinatal women experience common mental disorders such as anxiety and depression with substantial variations across settings (Fisher et al., 2012). Some of the variations are hypothesised to be due to differences in screening tools, for example the use of diagnostic tool versus self-reporting tools and the use of different cut-off points. For example, the reported prevalence of postnatal depression among adults in a peri-urban in South Africa was 38.8 % with EPDS cut-off point of 13 (Fisher et al., 2012; Omole & Phukuta, 2020) while a study of perinatal adults in Malawi reported a prevalence of 30.4% using Self-Reporting Questionnaire SRQ and 21% using the Structured Clinical Interview for DSM-IV (SCID) (Stewart et al., 2014). Evidence shows that adolescent mothers are more likely to experience common mental disorders compared with adult mothers (Beşenek & Gürlek, 2021; Field et al., 2020).The rate of postpartum depression in adolescent mothers is estimated between 14 and 53% worldwide (Dinwiddie et al., 2018; Swift et al., 2020) Furthermore, depressive symptoms persist in adolescent mothers for longer durations after delivery compared with adult mothers (Hymas & Girard, 2019).*  *The risk factors for postnatal depression in low and middle income countries include poverty due to socio-economic hardship, lack of emotional and practical support, intimate partner abuse, HIV status, inadequate social and familial support, unintended pregnancy and a negative societal perception of adolescent pregnancy (Baron et al., 2016; Beşenek & Gürlek, 2021; Field et al., 2020)* |
|  |  |
| The introduction section states the aim of the study as “aims to explore how social and cultural factors influence the mental health of adolescent mothers in rural Malawi,” while the objective in the abstract section reads, “To explore the perceived cultural and social factors that influence mental health among adolescent mothers during the postnatal period in rural Malawi.” These two statements are quite similar but not entirely aligned in terms of focus. The introduction suggests a broader scope of exploring actual social and cultural factors, while the abstract narrows it down to perceived factors, specifically during the postnatal period. There is a need to maintain consistency in the abstract, introduction, and research question. Methods | Thank you for your comment. The sentence in the introduction has been amended lines 105- 106. Now it reads  “a*ims to explore the perceived social and cultural factors that influence the mental health of adolescent mothers in rural Malawi”*  considering that the quality of evidence in a qualitative report is subjective. |
| It is suggested that the author could include the Theoretical framing as a part of the methods and not the introduction | Thank you for your comment. We have shifted this to methods |
| The facility is 128 enclosed by a brick fence, and just outside of the fence there is an open, crowded market with 129 many restaurants, some drug stores, pubs, and many herbalists selling different kinds of herbal 130 medicines.” If it is not connected with the study in any way then I would like to recommend the author remove irrelevant contents throughout the manuscript to keep it concise and specifically tailored to the stated research question. | Thank you for your comment. We have removed the description |
| In the method section, under Study Design the author has provided valuable information about the researcher’s background and its potential impact on the study. However, to enhance clarity and focus, consider streamlining this portion. For instance, while the researcher’s cultural background is crucial, details about their age and specific personal experiences might be less relevant. A more concise explanation emphasizing the researcher’s role and steps taken to ensure objectivity could be beneficial. This would help maintain the reader’s focus on the study’s design and methodology. | Thank you for your comment. We have removed the sentence on researcher’s experience. However, some information was included to demonstrate the researcher’s self-awareness to eliminate biasness |
| In the participants recruitment and sampling, it is mentioned that “participants were adolescent mothers aged 19 and younger from the catchment area of Mitundu Hospital”, it is unclear if this means those living near Mitundu Hospital or those who came for an OPD visit in Mitundu Hospital or the mothers inside inpatient department were also taken. In the next line, it is stated that “Recruitment occurred when mothers attended a clinic for postnatal check-ups, under-five clinics, and family planning clinics at Mitundu Rural Hospital.” It is recommended that the author remove the redundancy and unwanted explanation to avoid confusion | Thank you for your comment. We have removed “Mitundu Hospital” since mothers were recruited at the clinics which are within Mitundu catchment area and the clinics are operated by Mitundu Hospital. |
| Interested adolescents meeting the eligibility criteria were provided with an information sheet” What were the eligibility criteria? | Thank you for your comment, we have added the inclusion criteria line 143  *The study participants were adolescent mothers aged 19 and younger who were less than 12 months in the postnatal period and had the capacity to consent.* |
| Participants completed a survey and in-depth interview detailed in previous publications (Tembo et al., 2022; Tembo et al., 2023)” This statement suggests that this is a secondary paper. But in the introduction section or methodology, there is no mention of this case. For better transparency, it would be better if the author stated the nature of the primary study, how this research question was derived from this original study, and how it differs. | Thank you for your comment. This has been mentioned in the introduction specifically the last sentence on the aim lines 105-107  *This study forms part of a broader study (Tembo et al. 2022) and aims to explore the perceived social and cultural factors that influence the mental health of adolescent mothers in rural Malawi*.  Methods  This qualitative aspect was taken concurrently with the quantitative data collection, which has been published and referenced in this manuscript. This manuscript focuses only on qualitative data collection. |
| Were all 395 interviews? How many days does it take for the author to transcribe the interviews? | Thank you for your question. The transcribing took several months. There were variations in terms of content to transcribe, which meant some scripts took more hours and others were shorter. some interviews were brief because some participants were reserved and did not want to express more, and silence was also used as data. |
| The authors mentioned that NVivo version 11 was used. Were all of the coding and analysis done manually or with the use of NVivo | Coding was done using NVivo |
| Were the transcripts sent to the participants for their reflection | Thank you for your comment. Participants’ transcripts were not sent back to participants as it was difficult to follow them up. However, at the end of each interview we verified some important points with the mother. |
| **Result** |  |
| Suggest changing the heading Findings to Results | Thank you for your suggestion. This has been changed |
| Is there any specific reason to mention this in the result section “Three hundred and ninety- five interviews were conducted with adolescent mothers.” This is already stated in the method section | Thank you for your comment. We have deleted the sentence |
| With mean it is statistically sound to express the SD as well. What was the minimum and maximum age range of the adolescent mothers? |  |
| Table 2 ‘N’ should be noted as ‘n | Thank you. This has been corrected |
| In the overall result section, it felt like the stated themes, codes, and verbatim are not tailored to the specific research question i.e. what cultural and social factors influence mental health among adolescent mothers in rural Malawi? The result needed to be revised to make it concise and tailored to the stated research question.The presentation of themes in the results section could be clearer. Specifically, the "overarching theme awareness" and how it influences other themes, such as "relationships," "family support," and "rites of passage," needs more explanation. It would be helpful to explicitly describe how each of these themes interrelates with maternal mental health | Thank you for your comment. We have revised the results section to provide some linkages as below    Lines 224 -229  *The overarching theme of physical and mental health awareness provides context for the impact of social and cultural factors. Participants understanding of health reflects learnings and beliefs which are likely to have been influenced by family and cultural beliefs. Prior to discussing mental health, participants were asked about their understanding of health in general. Most participants demonstrated limited understanding of general health and did not discuss physical and mental health concurrently. Participants did however suggest ‘good health’ as being physically healthy and being free from sickness, having a good diet, having ‘enough blood’ and not experiencing any* pain.  Lines 267- 279  *Many participants reported that issues related to relationships in the context of marriage had impacted their psychological wellbeing during the perinatal period, with some discussing positive aspects of relationships while others described stressors. Most participants discussed the importance of having a partner. as a provider of personal and financial support. A few adolescent mothers who were single and unmarried wished they were married, especially if the father of the child did not wish to marry them. However, other single mothers lived with their parents and discussed being happy with this arrangement*  Line 371-374  *Unhappy marriages were mostly driven by forced marriages where some adolescent mothers experienced powerlessness by being coerced into marriages, which resulted in psychological abuse, financial stress and pressure to conform. These influenced chronic stress and poor mental health*  Line 394-400  *The impact of social and cultural influences on relationships of adolescent mothers impacted positive mental wellbeing for some while for others caused distress and unhappiness. Cultural beliefs impacted early marriage and for adolescents who became pregnant to marry. For some the stressors of being in a relationship exacerbated mental health issues, while for others this was a positive experience. For some remaining unmarried contributed to better wellbeing while for others, stressors around social expectations and financial pressures exacerbated mental health issues.*  Line 441-445  *Family support acted as a protective factor and contributed to enhanced wellbeing for adolescent mothers who received good support. These mothers benefited from the social and cultural influences whereby family members passed on parenting skills and were emotionally and financially supportive. However, rejection from the family negatively impacted participants social and emotional wellbeing* |
| The cultural context of the findings is not fully explored. The influence of specific cultural practices, beliefs, or norms related to motherhood, mental health, or adolescence in rural Malawi should be expanded upon. The connection between these practices and adolescent mothers’ mental health is essential and could strengthen the overall findings. | Thank you for your comment. The influence of the cultural aspect that was reported related to motherhood was mainly on the rituals, and we have provided a brief linkage to expand    Line 532-536  *The rituals, such as postnatal confinement, were often accompanied by increased social support and, therefore, reduced the burden of care while promoting the bonding of the infant with the mother, which was positive for good mental health. However, for other adolescent mothers, the superstitions and failure to adhere to rituals led to feelings of isolation, worry, guilt and fear which negatively impacted the adolescent's mental health* |
| While the quotes are compelling, their connection to the broader themes should be made clearer. Currently, they seem somewhat isolated from the discussion of themes. Each quote should be explicitly tied back to the theme or subtheme it supports. This would make the narrative flow more coherent and strengthen the findings. | Thank you for your comment. we have revised this section to make the narrative flow |
| It would be better to reflect some anonymous socio-demographic information of participants in the quotes, so the readers could get to explore the difference in the expression across age, married/unmarried status, level of literacy, and so on. | Thank you for your comment. We have added age and marital status to the quotes |
| The results section lacks an in-depth exploration of how specific social and cultural factors (e.g., rites of passage) affect mental health. The connection between these factors and adolescent motherhood is vital but not sufficiently detailed. | Thank you for your comment.  in this society or ethnic group, people are not expected to reveal their practices to strangers. As a result, most of their responses lacked depth and most of the participants avoided discussing more about it. This limited the scope of the interview. This was an example of the established connection  lines 505 -511    *Not having a partner to perform this ritual with or having a partner who was not trustworthy during the abstinence period made mothers fearful of negative consequences. Participants without partners were also concerned about the lives of their babies. Some adolescent mothers who did not follow these rituals feared losing their children due to illness. A few participants reported having been forced to sleep with another man if they did not have a husband to fulfil the ritual after postnatal abstinence, hence subjecting them to sexual abuse and associated mental health problems.* |
|  |  |
| This discussion provides a comprehensive exploration of the social and cultural factors that influence adolescent maternal mental health in rural Malawi, framed within the Socio-Ecological Model. But certain improvements can be made | Thank you for your comment |
| In the discussion, it is stated that “Within the overarching theme health and mental health awareness, individual ‘s understanding of health and mental health was found to be a characteristic that was influential in shaping adolescents’ understanding and perceptions of mental health problems and the behaviors related to seeking help.” However, in the result section it is not established that the awareness is connected with or is influencing the perception of mental health or the occurrence of the mental health outcome in the adolescent. | Thank you for your comment. We have revised the results section to provide a linkage as described above. |
| While the author references some studies from Nepal, Mexico, and Ethiopia, it would be useful to draw more detailed comparisons between the findings of the current study and other studies on adolescent mental health. | Thank you for your comment. The limitations of literature on studies around adolescent mental health made the discussion limited. However, I have added a citation from Rwanda study which was conducted among adolescent mothers    Lines 591-599  *These issues are similar to findings from a systematic review of psychosocial risk factors for perinatal depression among female adolescents and a Rwandan study among adolescent mothers n=120 (Niyonsenga & Mutabaruka, 2021; Recto & Champion, 2017), and complement quantitative findings from the current research project, which found adolescents who were in polygamous relations, forced marriages, lacked social support, experienced intimate partner violence and those whose parents were not involved in decisions regarding their care were more likely to report common mental disorders (Tembo et al., 2023, 2024)* |
| The author should consider including quantitative studies from these countries in the Introduction section to provide a clearer picture of the magnitude of maternal mental health issues. | Thank you for your comment. we have included some quantitative studies in the introduction |
| Additionally, in the Discussion section, the author could enhance their analysis by comparing their qualitative findings with cultural and social factors related to maternal mental health identified in past qualitative as well as quantitative research. This will help contextualize the current study's findings within the broader body of research. | Thank you, we have added some information from of the quantitative findings line 612 -615  *These issues are similar to findings from a systematic review of psychosocial risk factors for perinatal depression among female adolescents and a Rwandan study among adolescent mothers n=120 (Niyonsenga & Mutabaruka, 2021; Recto & Champion, 2017), and complement quantitative findings from the current research project, which found adolescents who were in polygamous relation, forced marriages, lacked social support, experienced intimate partner violence and those whose parents were not involved in decisions regarding their care were more likely to report common mental disorders (Tembo et al., 2023, 2024)* |
| In terms of Ethical considerations, it is unclear how confidentiality and privacy were maintained. As for the minor's consent, it is mentioned that legal guardians witnessed the adolescent mother’s consent, were the interviews with the adolescent mothers taken in the presence of their mothers and/or legal guardians? It is mentioned that “Any woman who reported suicidal ideation was counseled and offered a referral for clinical mental health assessment and treatment” but throughout the result section the information regarding suicidal ideation seems to be missing. | Thank you for your comment.  Adolescents were not interviewed with their mother as they were considered as mature minors except a few who were 14 years and came in the company of their mother. Suicide ideation was not a theme however as described above this study was part of the survey which had quantitative questions to identify potential risk for postnatal depression and anxiety. Within the questions on depression were questions on self-harm. This is how some mothers who were at risk were counselled and referred. However, the qualitative suicide did not come out as a theme |

Baron, E. C., Hanlon, C., Mall, S., Honikman, S., Breuer, E., Kathree, T., Luitel, N. P., Nakku, J., Lund, C., Medhin, G., Patel, V., Petersen, I., Shrivastava, S., & Tomlinson, M. (2016, 2016/02/16). Maternal mental health in primary care in five low- and middle-income countries: a situational analysis. *BMC Health Services Research, 16*(1), 53. <https://doi.org/10.1186/s12913-016-1291-z>

Beşenek, A. M., & Gürlek, B. (2021). New aspects of being an adolescent mother: Comparison of psychosocial features between adult and adolescent mothers.

Dinwiddie, K., Schillerstrom, T. L., & Schillerstrom, J. E. (2018). Postpartum depression in adolescent mothers. *Journal of Psychosomatic Obstetrics & Gynecology, 39*(3), 168-175.

Field, S., Abrahams, Z., & Honikman, S. (2020). Adolescent mothers: A qualitative study on barriers and facilitators to mental health in a low-resource setting in Cape Town, South Africa. *African Journal of Primary Health Care & Family Medicine, 12*, 1-9. <http://www.scielo.org.za/scielo.php?script=sci_arttext&pid=S2071-29362020000100036&nrm=iso>

Fisher, J., Cabral de Mello, M., Patel, V., Rahman, A., Tran, T., Holton, S., & Holmes, W. (2012, Feb 1). Prevalence and determinants of common perinatal mental disorders in women in low- and lower-middle-income countries: a systematic review. *Bull World Health Organ, 90*(2), 139g-149g. <https://doi.org/10.2471/blt.11.091850>

Hymas, R., & Girard, L.-C. (2019). Predicting postpartum depression among adolescent mothers: A systematic review of risk. *Journal of affective disorders, 246*, 873-885. <https://www.sciencedirect.com/science/article/pii/S0165032718315921?via%3Dihub>

Niyonsenga, J., & Mutabaruka, J. (2021, 2021/10/02). Factors of postpartum depression among teen mothers in Rwanda: a cross- sectional study. *Journal of Psychosomatic Obstetrics & Gynecology, 42*(4), 356-360. <https://doi.org/10.1080/0167482X.2020.1735340>

Omole, O. B., & Phukuta, N. S. J. (2020). Prevalence and risk factors associated with postnatal depression in a South African primary care facility. *African Journal of Primary Health Care and Family Medicine, 12*(1), 1-6. <https://doi.org/doi:10.4102/phcfm.v12i1.2538>

Recto, P., & Champion, J. D. (2017, Aug). Psychosocial Risk Factors for Perinatal Depression among Female Adolescents: A Systematic Review. *Issues Ment Health Nurs, 38*(8), 633-642. <https://doi.org/10.1080/01612840.2017.1330908>

Stewart, R. C., Umar, E., Tomenson, B., & Creed, F. (2014, Apr). A cross-sectional study of antenatal depression and associated factors in Malawi. *Arch Womens Ment Health, 17*(2), 145-154. <https://doi.org/10.1007/s00737-013-0387-2>

Swift, E. R., Pierce, M., Hope, H., Osam, C. S., & Abel, K. M. (2020). Young women are the most vulnerable to postpartum mental illness: A retrospective cohort study in UK primary care. *Journal of affective disorders, 277*, 218-224. <https://www.sciencedirect.com/science/article/pii/S0165032720326124?via%3Dihub>

Tembo, C., Portsmouth, L., & Burns, S. (2023). Postnatal depression and its social-cultural influences among adolescent mothers: A cross sectional study. *PLOS Global Public Health, 3*(6), e0002025. <https://doi.org/10.1371/journal.pgph.0002025>

Tembo, C., Portsmouth, L., & Burns, S. (2024, 2024/08/05). Cultural and social factors associated with generalized anxiety disorder among adolescent mothers during the postpartum period in Malawi: a cross-sectional survey. *BMC Global and Public Health, 2*(1), 50. <https://doi.org/10.1186/s44263-024-00080-3>
